# Epstein-Barr Virus-Positive Natural Killer/T-Cell Lymphoma

**DOI:** 10.3389/fonc.2019.00386

**Published:** 2019-05-14

**Authors:** Qingqing Cai, Jun Cai, Yu Fang, Ken H. Young

**Affiliations:** ^1^State Key Laboratory of Oncology in South China, Collaborative Innovation Center for Cancer Medicine, Sun Yat-sen University Cancer Center, Guangzhou, China; ^2^Department of Medical Oncology, Sun Yat-sen University Cancer Center, Guangzhou, China; ^3^Department of Hematopathology, The University of Texas MD Anderson Cancer Center, Houston, TX, United States

**Keywords:** extranodal natural killer/T-cell lymphoma, nasal type (ENKL), Epstein-Barr virus, molecular pathogenesis, diagnosis, treatment, prognosis

## Abstract

Extranodal natural killer/T-cell lymphoma, nasal type (ENKL), is a rare malignancy of Non-Hodgkin lymphoma characterized by an aggressive clinical course and poor prognosis. It shows strong association with Epstein-Barr virus infection and occurs more commonly in Asia and Latin America. Various genetic alterations have been identified in ENKL by gene expression profiling and sequencing techniques. The frequent deletion of chromosome 6q21 was reported to lead to the silence of several tumor suppressor genes. Also, there have been novel genetic mutations that were recently uncovered and were found to frequently activate several oncogenic pathways, including the JAK/STAT, NF-κB, and MAPK pathways. Besides, we believe that deregulated single genes and epigenetic dysregulation might be relevant to the mechanism of this disease and thus, may have the potential to shed lights on the development of new therapeutic strategies. The consensus on the standard treatment for ENKL has not yet been currently established. For localized ENKL patients, radiotherapy with concurrent chemotherapy and sequential patterns of chemotherapy and radiotherapy are recommended as first-line therapy. As for advanced or relapsed/refractory ENKL patients, the application of non-anthracycline-containing regimens have significantly improved the clinical outcome, contributing to higher response rate, longer overall survival and progression-free survival. Hematopoietic stem cell transplantation is widely recommended for consolidation after a complete remission or partial remission has been achieved. The anti-programmed death 1 antibody, an immune checkpoint inhibitor, has demonstrated favorable results in treating relapsed or refractory ENKL. Of the current ENKL treatment, researchers are still striving to validate how radiotherapy and chemotherapy should be optimally combined and which of the non-anthracycline-containing regimens is superior. In this review, we summarize the main genetic alterations frequently found in ENKL and their role in providing new insights into the therapeutic targets of this disease, and highlight the recent findings regarding new biologic markers, novel therapeutic strategies applied to this intriguing neoplasm.

## Introduction

Extranodal natural killer/T-cell lymphoma, nasal type (ENKL) is a rare and distinct malignancy with an aggressive clinical course. Its lesions are predominantly present in the upper aerodigestive tract (UADT). Since the neoplasm can destroy the midline facial structures, the disease used to be known as lethal midline granuloma (1–3). ENKL is strongly associated with Epstein–Barr virus (EBV) infection and occurs more commonly in Asia and Latin America. Vast majority of ENKL express CD56, CD2, cytoplasmic CD3ε and cytotoxic markers, and lacked the rearrangement of T-cell receptor gene, which supports a natural killer cell derivation. Rare cases of T cell derivation with a cytotoxic phenotype have also been described and are included in this subtype (4, 5).

## Pathogenic Mechanisms of Epstein-Barr Virus-Positive NK/T-Cell Lymphoma

### Epstein-Barr Virus Gene Expression

Epstein-Barr virus (EBV) resides in the B-cells, T-cells, NK-cells, epithelial cells and mesenchymal cells of the infected individuals (6). The mechanism by which B cells are infected has been well-elucidated, but the mode of infections in other cell types is less well-known. During the infective period, EBV transforms the host cells from a resting state to a malignant activated state, and facilitates the oncogenesis of nasopharyngeal carcinoma and lymphomas, such as ENKL, Burkitt lymphoma, diffuse large B-cell lymphoma (DLBCL) and classic Hodgkin's lymphoma.

In ENKL, it is not clear whether the EBV genomes passage is as an episomal form or integrated into host genome. However, using high-throughput sequencing, our recent study identified frequent focal EBV genome deletions and integrated EBV fragments in the host genome in ENKL (7). Once the B cell is infected EBV, the virus will induce expression of numerous genes (the viral as well as the host gene) to evade the innate and acquired immune system, and facilitate transformation of B cells through a variety of mechanisms. During the primary infection, EBV undergoes a lytic stage and latent stage and then establishes a permanent reservoir of memory B-cells. The lysis phase is characterized by massive viral products in the host cells due to the lytic gene expression. While various patterns of latency type are observed in different lymphomas, such as latency type I in Burkitt lymphoma, latency type II in classic Hodgkin lymphoma and latency type III in post-transplant lymphoproliferative disorders of B cell type. EBV expresses viral-associated latent proteins including EBV-determined nuclear antigen (EBNA) and latent membrane proteins (LMP-1,−2a, and−2b), which are contribute to the division of the viral genome during cell division and differentiation of EBV-infected cells to long-lived memory cells (8–11). Among all these latent products expressed in ENKL cells, LMP-1 is the main oncogenic protein which inhibits apoptosis and promotes cell-cycle progression, proliferation, migration, and invasion. Further, LMP-1 upregulate the expression of survivin, myc, soluble IL-2 receptor alpha (IL-2R α), and programmed death protein ligand 1 (PD-L1) through multiple downstream signaling pathways, including the MAPK/ERK1/2, JAK/STAT, NF-κB, and PI3K/Akt pathways (12–17).

### Genetic Susceptibility

Genome-wide association study of ENKL has identified several single-nucleotide polymorphisms (SNPs) directly related to ENKL susceptibility. Among those SNPs, rs9277378^*^A risk allele, which is located within the *HLA-DPB1* gene on chromosome 6, was found to have the strongest association for an increased risk of ENKL (18). Deletion mutation of *RASGRPI*, a T lymphocyte-specific nucleotide exchange factor of T-cells for proliferation and anti-EBV immune responses, may also result in a high susceptibility to EBV infection and EBV-driven lymphoproliferative disorders (19). Moreover, numerous genes (such as ITK, SH2D1A, CD27, CORO1A) which affect T cells, NK cells and their interaction with antigen presenting cells, as well as EBV infected B cells and macrophages, also increase susceptibility to ENKL (20).

### Chronic Inflammation and Lymphoma Microenvironment

ENKL initiates chronic inflammatory responses in which immune-associated pro-inflammatory and anti-inflammatory cytokines are overexpressed (21). The interactions between monocytes and membrane-bound IL-15 increases the expression of LMP1 and promotes the proliferation of ENKL cells (22). Simultaneously, PD-L1 and PD-L2 may be upregulated by inflammatory cytokines, of which interferon-γ may play a key role (23). The presence of lymphoma-associated hemophagocytic syndrome (LAHPS) during the progression of lymphoma may indicate an extremely poor outcome. A recent study has identified a hotspot gene mutation of evolutionarily conserved signaling intermediate in the Toll pathway (*ECSIT -T419C*). Tumor cells harboring the *ECSIT-T419C* mutation facilitate a massive secretion of pro-inflammatory cytokines (including TNF-α and IFN-γ) and trigger a hyperinflammation, thereby promoting LAHPS in ENKL (24).

### Molecular Pathogenesis

#### Chromosomal Abnormalities

**Figure 1:**
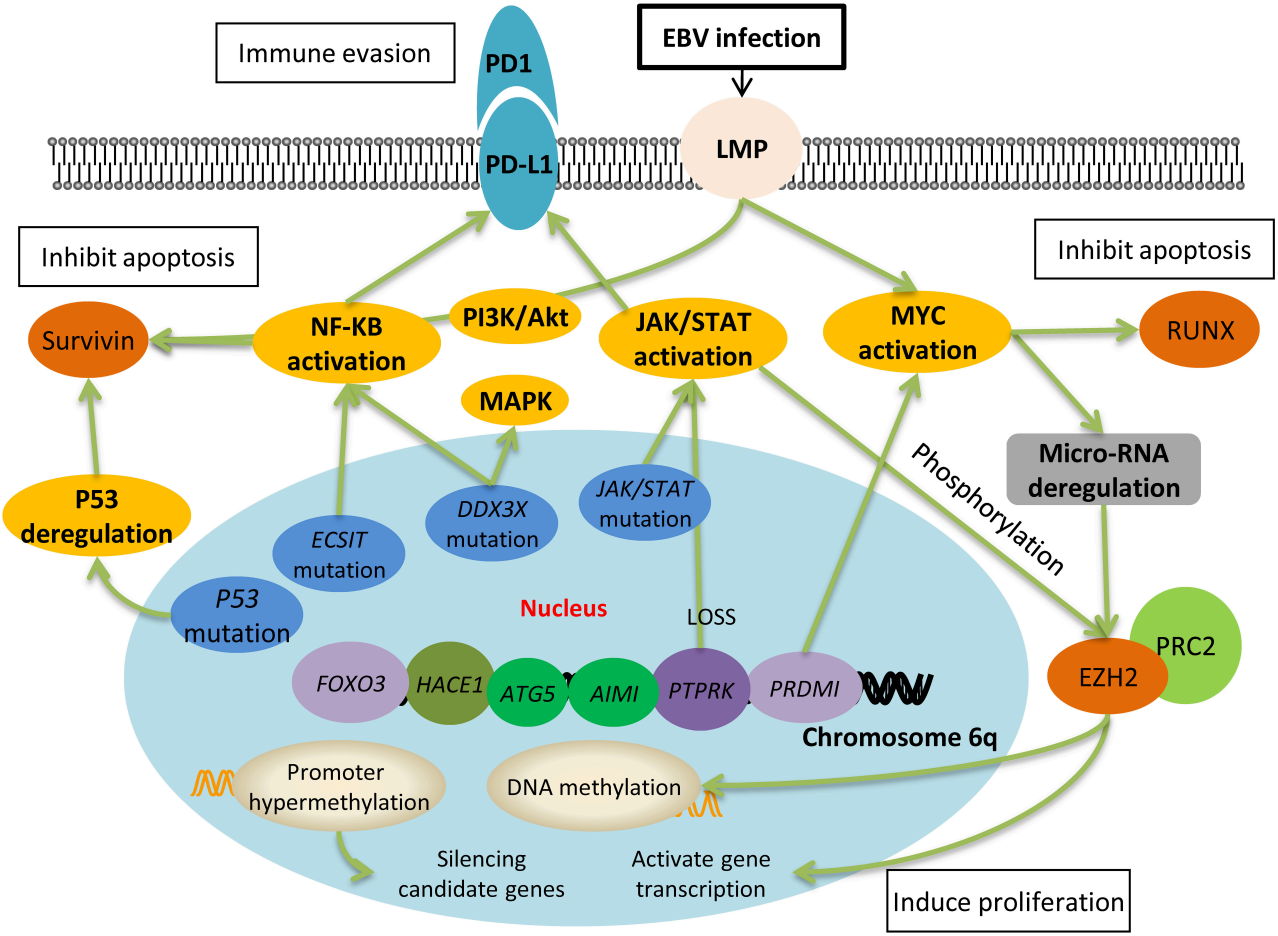
Molecular pathogenesis of Epstein-Barr virus-positive NK/T-cell lymphoma. EBV latent membrane protein-1 (LMP-1) activates the downstream signaling pathways, one of which is the NF-κB pathway. Together with PI3K/Akt pathways, NF-κB leads to the upregulation of survivin, which further inhibits cell apoptosis. Activation of JAK/STAT is associated with the upregulation of EZH2, which results in DNA methylation and gene transcription, and ultimately induces cell proliferation. Both the NF-κB pathway and JAK/STAT pathway upregulates the expression of PD-L1 on the surface of lymphoma cells and they escape from the immune surveillance of activated T cells. The deletion of chromosome 6q silences many tumor suppressor genes, such as *PTPRK* and *PRDMI*, which directly result in the activation of JAK/STAT and myc pathways. Moreover, gene mutation including P53, *ECSIT, DDX3X* are also involved in the oncogenesis of ENKL through the pathways mentioned above.

Genome-wide studies have reported several chromosomal abnormalities in ENKL. The most typical one is the deletion in the chromosome 6 region, which directly lead to the silence of several tumor suppressor genes coding for *PRDM1, FOXO3, ATG5, AIM1*, and *HACE1* (Figure 1). Moreover, it has been reported that the re-expression of *FOXO3* and *PRDM1* suppress the proliferation of ENKL cells (14, 25–27); suggesting their involvement in oncogenesis. However, further study failed to prove the role of *HACE1* as a tumor suppressor in ENKL (26).

In non-Hodgkin's lymphoma, deletion in chromosome 6 region has been well-studied in B cell lymphomas and proved to be the most common secondary aberration including tumor suppressor genes' loss and oncogenes' amplification (28). However, how the deletion in chromosome 6 promote ENKL development is puzzled and whether it represents a primary or secondary event in ENKL is debatable, and more studies are needed to elucidate this alteration and the mechanism behind it.

#### Gene Mutations

The discovery of frequent gene mutations in ENKL has somehow revealed its underlying molecular mechanism and has provided promising molecular targets for this disease. Exome sequencing has shown that the *DEAD-BOX(DDX)3X* mutation was present in ~50% of all ENKL, inhibited RNA-unwinding activity, and deregulated suppressive effects on NF-κB and MAPK pathways. Located on the sex chromosomes X, the *DDX* gene acts as a tumor suppressor. It encodes RNA helicases, a large family of proteins that unwind the double stranded RNA and take part in multiple biological processes within the NK cells. Thus, mutations disrupting those crucial functions will consequently promote cell growth and tumor progression (29–31).

Mutation of *ECSIT-T419C* encode a V140A variant of *ECSIT*, which activate the NF-κB signal pathway by binding with the S100A8 and S100A9 heterodimer and enhancing the activity of NADPH oxidase, and thus play a key role in inflammatory response of LAHPS (24).

#### Aberrant Signaling Pathways

Several studies have focused on the functional alteration of Janus kinase (JAK) and the downstream molecular signal transducer and activator of transcription (STAT) pathway, which predominantly mediate the IL-2 receptor signaling, contributing to the tumor cell development and invasive phenotype of ENKL (32). Acquired mutation (*A573V, V722I, A572V, and A573V*) in the JAK3 pseudokinase domain resulted in constitutive JAK3 activation (33, 34). Ectopic expression of *STAT3* missense single-nucleotide variants (SNVs) and *STAT5B* missense mutations were found to be associated with increased phosphorylated protein, promoting the growth of transduced cell lines or primary human NK cells (35). Additionally, loss expression of the receptor-type tyrosine-protein phosphatase kappa (PTPRK), the only protein tyrosine phosphatase at 6q that contains a STAT3-specifying motif, may directly lead to STAT3 activation and over-expression in ENKL (36).

**Figure 2:**
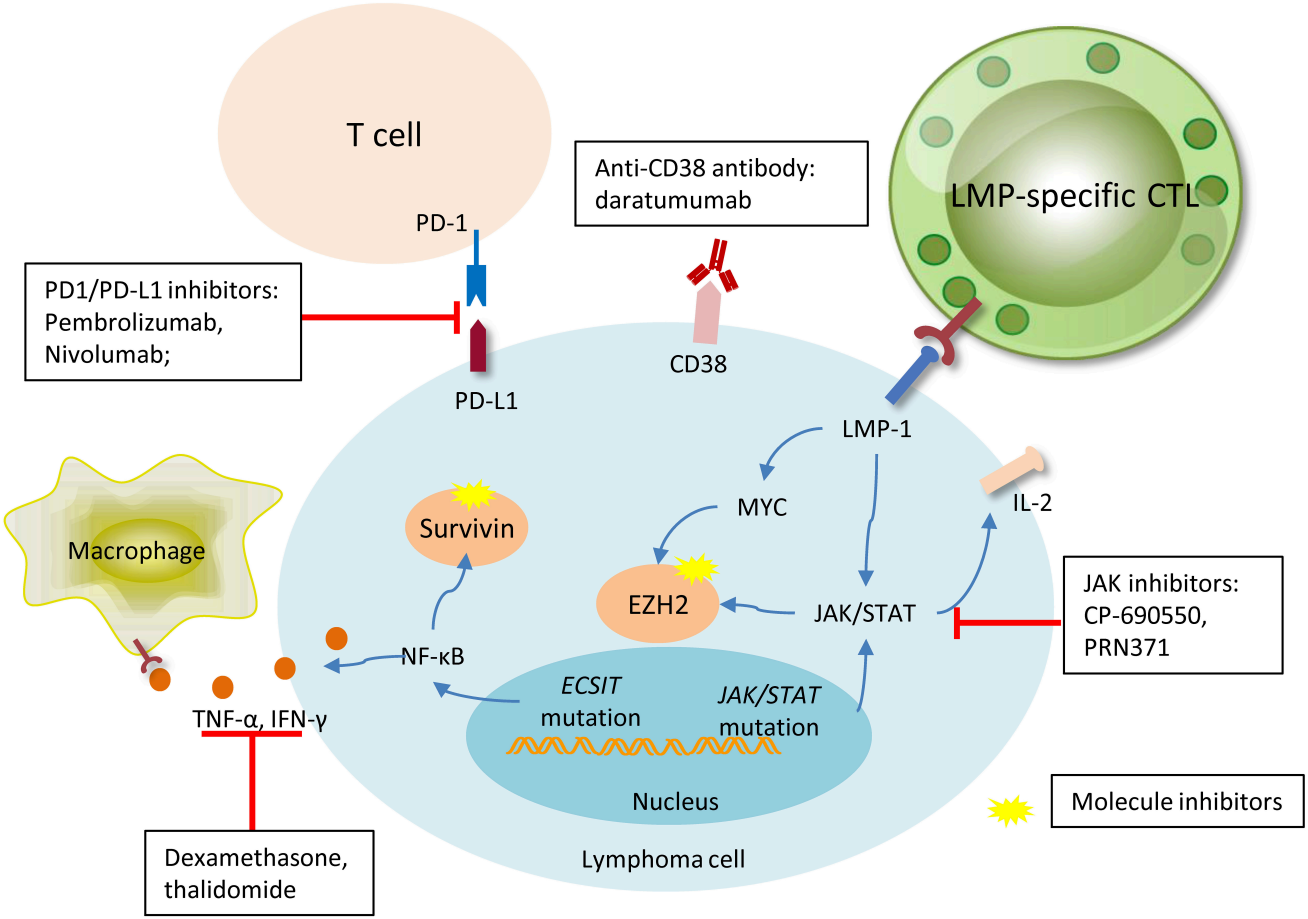
Promising new drugs or treatment strategies in NK/T cell lymphoma and their target. Pembrolizumab and nivolumab are both anti-PD1 antibodies, they were designed to target PD1 on the microenvironment T-cells, interrupting the connection of PD1 and PDL-1, and inhibiting activation. Daratumumab, a novel antibody targeting CD38 on the membrane of lymphoma cell, has shown particular efficacy in one case. LMP-specific CTLs are produced against lymphoma cells with membrane expression of LMP, and sequentially kill tumor cells. Signal pathways aberration and gene dysregulation are universally involved in lymphomagenesis, thus making molecule inhibitors an attractive target. JAK inhibitors, surviving, and EZH2 blocades have shown efficacy in some pre-clinical trials with favorable outcomes. The immunosuppressive agents, dexamethasone, and thalidomide, can efficaciously inhibit the TNF-α and IFN-γ released by lymphoma cells due to the *ECSIT* mutation, and relieve ENKL-associated hemophagocytic syndrome.

Network and signaling pathway analysis also recognized activated NOTCH, p53, NF-κB, PDGF, AKT, and MAPK/ERK1/2 pathways that are differentially expressed between ENKL and normal NK cells (Figure 1) (14, 37). Drugs targeting these activated pathways have made some progress in terms of medical development and have shown practical therapeutic potential in the clinical treatment (Figure 2).

#### Deregulated Single Genes

It is well-defined that EZH2 overexpression is related to the tumorous invasive growth and poor clinical outcomes in several types of cancers. Known to be the key enzymatic component of polycomb repressor complex 2 (PRC2), EZH2 depends on PRC2 to establish an oncogenic function, which directly controls DNA methylation and leads to the silence of a magnitude of genes as a transcriptional repressor (38, 39). Whereas, in the recent years, there has been increasing literatures showing that EZH2 not only acts as a “transcriptional repressor” but also as a “transcriptional activator.” In ENKL, EZH2 is independent on the PRC2's enzymatic activity and shows a non-canonical function of directly activating gene transcription, which in turn transform EZH2 from a transcriptional repressor into a “transcriptional activator.” It has also been demonstrated that EZH2 phosphorylation was regulated by the JAK3 pathway for inducing its dissociation from PRC2 (40), and the upregulation of EZH2 is reported to be induced by myc's suppression effect on its regulatory microRNAs in ENKL (39).

Runt-domain transcription factor (RUNX3), not only shows oncogenic properties implicated in several major developmental pathways, but also acts as a tumor suppressor that increase apoptosis and reduce cell proliferation (41, 42). Recent studies have found that the overexpression of RUNX3 in ENKL, and myc transcriptional regulated RUNX3 by binding with it, sequentially resulted in increased proliferation of tumor cells. Satisfactorily, cell growth could be effectively inhibited by a small-molecule *MYC* inhibitor which caused significant downregulation of myc and RUNX3 and thus, providing new therapeutic implications for clinical application (43).

#### Epigenetic Dysregulation

The discovery of frequent distal DNA hypomethylation and promoter hypermethylation, which may be responsible for suppressor genes being silenced, has highlighted the prominent role of epigenetic deregulation in ENKL (44). Epigenetic regulators mutations of BCOR and MLL2 were frequently seen in ENKL, playing a key role in oncogenesis as tumor suppressor genes (45). In addition, miRNAs such as miR-101, miR26b, miR-26a, miR-28-5, and miR-363, were found to be dramatically downregulated in ENKL compared to normal NK cells, to increase the expression of several genes involved in oncogenesis, and to disturb cell growth (46). All the molecular alterations are summarized in Table 1 below.

**Table 1:** Molecular pathogenesis of Epstein-Barr virus-positive NK/T-cell lymphoma.

**Molecular pathogenesis**	**Genetic alterations**	**Highlights**	**Target therapy**
Chromosomal 6 abnormalities	*PRDM1* (47, 48)	Acts as both potential tumor suppressor and promoter. Hypermethylation of *PRDM1* downregulated its expression and involved in the oncogenesis of ENKL.	
	*FOXO3* (25)	A tumor suppressor belonging to the fork-head family.	
	*PTPRK* (36)	The only protein tyrosine phosphatase at 6q that contains a STAT3-specifying motif. Loss of expression of *PTPRK* directly led to STAT3 activation and tumor progression in ENKL.	
	*HACE1* (26)	A tumor suppressor, but it is not directly involved in the ENKL pathogenesis.	
Gene mutations	*DDX3X* (30)	A tumor suppressor gene. *DDX3X* mutants reduced RNA-unwinding activity and deregulated suppressive effects on the NF-κB and MAPK pathways.	
	*ECSIT* (24)	A gene associated with LAHPS. *ECSIT-T419C* mutants activated NF-κB signal pathway by binding with S100A8 and S100A9 heterodimer and enhanced the activity of NADPH oxidase inflammatory response.	Dexamethasone and thalidomide
Aberrant signaling pathways	JAK/STAT (33–35, 49, 50)	Acquired mutations (A573V, V722I, A572V, A573V, H583Y, and G589D) in the JAK3 pseudokinase domain resulted in constitutive JAK3 activation. STAT3 missense single-nucleotide variants (S614R, G618R, and A702T) and STAT5B missense mutation were located in the SH2 domain, which was critical for STAT activation and further promoted growth, survival and invasiveness of tumor cells. Constitutive JAK3 phosphorylation on tyrosine 980 was also involved in JAK3 activation.	JAK inhibitors CP-690550, PRN371
	NF-κB (24, 30, 51)	NF-κB was highly expressed in ENKL compared with normal NK cells. It was the key signaling pathway that was implicated in several genetic alterations, such as the overexpression of survivin, *DDX3X* mutants and LAHPS.	Bortezomib
Deregulated single genes	Survivin (12, 37, 52)	Overexpression of survivin was detected in 97% of cases. And LMP-1 was proved to upregulate the expression of survivin through NF-κB and PI3K/Akt signaling pathways.	Terameprocol (EM-1421)
	Myc (37, 40, 43)	Myc was highly expressed in the NK cell lines and tumor samples. Activation of myc was related to the upregulation of EZH2 and the overexpression of RUNX 3.	
	EZH2 (39, 40, 53)	EZH2 is a H3K27 methyltransferase that directly controls DNA methylation and silences several genes as a transcriptional repressor. It is also a “transcriptional activator” for driving oncogenesis. In ENKL, JAK3 could lead to the phosphorylation of EZH2 and resulted in the upregulation of several genes which were involved in DNA replication, cell cycle, and invasiveness. Also, the expression of EZH2 could be upregulated by myc by inducing repression of its regulatory microRNAs.	EZH2 inhibitor GSK126
	RUNX3 (42, 43)	RUNX3 only shows oncogenic properties, but also acts as a tumor suppressor. myc transcriptional regulated RUNX3 by binding activity with it in NKTL. Inhibition of *MYC* could effectively inhibit RUNX3, following by increased apoptosis and reduced cell proliferation.	
Epigenetic dysregulation	Hypermethylation (44)	Promoter hypermethylation is responsible for several suppressor genes being silenced, and those probably involved in ENKL are BCL2L11 (BIM), DAPK1, PTPN6 (SHP1), TET2, SOCS6, and ASNS.	
	MicroRNA (46, 54, 55)	MicroRNAs, such as miR-101, miR26b, miR-26a, miR-28-5, and miR-363, were found dramatically downregulated in ENKL compared to normal NK cells. They functioned by regulating the expression of their predicted target genes. MiR-15a was reported to be downregulated by LMP-1. Besides, miR-146a could downregulate NF-κB activity by targeting TRAF6 and function as a tumor suppressor, which sequentially promote cell proliferation and predict poor prognosis.	

## Diagnosis of Epstein-Barr Virus-Positive NK/T-Cell Lymphoma

### Clinical Features and Stage Stratification

ENKL predominantly occurs in the extranodal sites, and damages the midline facial structures and other sites including the orbit, salivary glands and paranasal sinuses (1–3). It is common to observe systemic symptoms (B symptoms: fever, weight loss and night sweats) as well as lymphoma-associated hemophagocytic syndrome in advanced cases (56, 57).

Dedicated computed tomography (CT) and magnetic resonance imaging (MRI) scans are universally used for the assessment of nasal cavity, hard palate, anterior fossa and nasopharynx. To determine the optimal management of those patients, the Ann Arbor staging system was widely used based on the CT and PET/CT imaging of the lymphoma location (same side of diaphragm, opposite side of diaphragm, extra-nodal, etc.) (58). The imaging alteration together with circulating EBV-DNA after treatment are efficient methods of response assessment (59). PET/CT is wildly used for response assessment currently and the 5-point Deauville scale for response assessment using FDG-PET was incorporated into the Lugano classification criteria in 2011 (58).

### Diagnosis and Differential Diagnosis

Histologically, ENKL is frequently characterized with local angio-invasion and/or angio-destruction, causing progressive necrotic ulceration. Neoplastic cells are intermediate to large cell in size positive for surface CD2, CD56 and cytoplasmic CD3ε, but negative surface CD3. Cytotoxic markers for ENKL diagnosis include TIA-1, granzyme B, T-cell intracellular antigen 1 and perforin on tumor cells (5). Circulating EBV-DNA, a biomarker of tumor load, as well as the EBV-encoded RNA (EBER) by *in situ* hybridization are also essential biomarkers for diagnosis (59, 60).

The expression of CD3ε+, cytotoxic molecules and Epstein-Barr virus must be present for the diagnosis of ENKL based on the current WHO classification criteria (5). The differential diagnosis of ENKL can be judged by the common location of extranodal sites especially upper aerodigestive tract, the absence of surface CD3, CD5, TCR receptor, and elevated EBV-DNA level. It should be distinguished from other EBV-related hematological disorders, such as acute EBV-associated hemophagocytic lymphohistiocytosis (HLH), nodal EBV positive peripheral T cell lymphoma (PTCL), EBV-associated diffuse large B cell lymphoma and aggressive NK cell lymphoma.

### Prognosis, Biomarkers, and Risk Stratification

Previous prognostic models for risk stratification included the International Prognostic Index (IPI), the Korean Prognostic Index (KPI) and prognostic nomogram, all of which were based on treatments using primarily CHOP or CHOP-like regimens (61–63). As the treatment for this disease has changed from conventional anthracycline-based chemotherapy to non-anthracycline-based chemotherapy, a new prognostic model called prognostic index of natural killer lymphoma (PINK) was established. Consisting of four independent prognostic factors: age >60 years, stage III/IV disease, non-nasal type and distant lymph-node involvement, PINK provides a more accurate prediction of survival of ENKL compared with the previously available models. At the same time, it was the first time to integrate quantifiable circulating EBV-DNA into a prognostic index for natural killer cell lymphoma–Epstein-Barr virus (PINK-E) (64).

A multivariate study consisting of localized ENKL patients who received radiotherapy-DeVIC revealed that elevated pretreatment soluble IL-2 receptor could also be an independent prognostic factor for poor overall survival (OS) and progression-free survival (PFS) (65). Besides, for localized ENKL patients who have received radiotherapy (RT), the assessment of their primary tumor invasion (PTI) and regional lymph node (LN) spread based on MRI are crucial prognostic factors for predicting their survival outcomes, and are considered when defining the clinical target volume (CTV) (66). Also, a high concentration of serum soluble PD-L1 and a high expression of PD-L1 in tumor specimens were also found to be independent adverse predictors for patients with stage I~II ENKL (15, 67).

Other potential prognostic factors, including elevated fasting blood glucose (FBG, >100 mg/dl at diagnosis) (68), decreased total protein (TP, < 60 g/L) (69) and non-O blood type (70), may predict lower response and worsen survival in ENKL patients. CD30, a target of anti-CD30 antibody for refractory Hodgkin lymphoma patients with strong expression of CD30, was detected in 47% of ENKL patients in a retrospective study, however, it failed to show any prognostic significance (71).

## Treatment of Epstein-Barr Virus-Positive NK/T-Cell Lymphoma

### Stage I/II NK/T-Cell Lymphoma

It is commonly recognized that RT is the most effective treatment modality in terms of locoregional control (LRC) for localized ENKL patients (72, 73). Even for patients who have achieved a complete response (CR) after chemotherapy, they still show higher survival rates with RT than without RT. A dose of 50Gy is established as the optimal dose for localized ENKL patients (74). A retrospective study which investigated the effect of dose-dependent RT on long-term survival in localized ENKL patients demonstrated that high-dose RT (≥50 Gy) significantly exhibited better 5-year LRC (85 vs. 73%), PFS (61 vs. 50%), and OS (70 vs. 58%) as compared to low-dose RT (< 50 Gy). High-dose RT was also observed to dramatically lower the risk of locoregional recurrence (73). However, a subset of patients who receive RT alone still face local recurrence and systemic failure.

The outcome of conventional chemotherapy of anthracycline-based regimens such as CHOP (cyclophosphamide, doxorubicin, vincristine, and prednisone) are unsatisfactory because of natural killer cells' high expression of the P-glycoprotein, which is encoded by multidrug resistance (MDR)-1 gene and results in resistance to anthracycline (75). Since the early 2000s, a mass of clinical trials was conducted to define the best approaches to treat ENKL patients. Accordingly, non-anthracycline-containing regimens showed outstanding efficacy.

A retrospective study has found out risk-adapted therapeutic strategies for localized ENKL: RT alone for low-risk patients and RT consolidated by chemotherapy for high-risk patients (76). Besides, a recent retrospective study aimed to define the role of sequential RT in combination with chemotherapy in comparison with concurrent chemoradiotherapy (CCRT) in localized ENKL showed comparable results. Treatment included single patterns (chemotherapy or RT), sequential patterns (chemotherapy followed by RT or RT followed by chemotherapy) and concurrent patterns (CCRT or CCRT followed by chemotherapy). It turned out that sequential patterns and concurrent patterns showed similar outcome. Both of them dramatically changed the clinical management of ENKL and were recommended as first-line therapy for localized ENKL patients (77). In conclusion, either sequential or concurrent chemotherapy with RT is the preferred treatment approach for early-stage ENKL and they show comparable efficacy in ENKL. Also, RT is only acceptable if the patient is unfit for combination chemotherapy.

#### Sequential Patterns

The sandwich protocols, which consist of sequential RT after an initial induction chemotherapy followed by further consolidation of chemotherapy, has also achieved comparable efficacy in contrast to concurrent patterns. In one study, it was observed that after 26 patients completed the sandwich LVP (L-asparaginase, vincristine and prednisone) with RT, they had an overall response rate (ORR) of 89%, which included 80.8% of the patients achieving a CR and 8% achieving a partial response (PR). With a median follow-up of 27 months, the 2-year OS and PFS rates were 89 and 81%, respectively. During this study, only 2 patients experienced grade 3 leukocytopenia (78). In another study, the regimen GELOX (gemcitabine, oxaliplatin, and L-asparaginase) was prescribed as a sandwich protocol to 27 patients with localized ENKL. At the end of the treatment, the observed ORR was 96%, including 74% with CR and 22% with PR. At a median follow-up of 27 months, the 2-year OS and PFS rates were both 86%. Grade 1–2 toxicities were common but grade 3–4 toxicities were few during this treatment (79).

In a retrospective study, sequential patterns of RT followed by GDP (gemcitabine, dexamethasone and cisplatin) regimen was applied to 44 patients with newly-diagnosed localized ENKL. After the completion of GDP chemotherapy, 8 patients with PR to former RT achieved CR, and the ORR was 95%. With a median follow-up of 38 months, the 3-year OS rate and PFS rates were 85 and 77%, respectively (80).

#### Concurrent Chemoradiotherapy

Localized ENKL patients were treated with RT (50 Gy) concurrently with the recommended dose of DeVIC (dexamethasone, etoposide, ifosfamide, and carboplatin) in a phase I/II study, and the ORR was 81%. With a median follow-up of 32 months, the 2-year OS rate reached 78%, compared favorably with the historical control of RT alone (45%) (81). Another study retrospectively analyzed 150 localized ENKL patients who also received RT concurrently with DeVIC and demonstrated an observed 5-year OS and PFS rate of 72 and 61%, respectively. Adverse effect of the RT-DeVIC regimen were comparable in these two studies, and grade 3 and 4 leukopenia and neutropenia, grade 3 mucositis relating to radiation were the most common toxicity (65).

CCRT (40–52.8 Gy of radiation and cisplatin) followed by three cycles of VIPD (etoposide, ifosfamide, cisplatin, and dexamethasone) was tested in a phase II trial for patients with stage I/II ENKL patients. The ORR and CR rate were 83 and 80%, respectively, and their estimated 3-year OS and PFS rates were 86 and 85%, respectively. Grade 4 neutropenia occurred in 12 of the 29 patients even though doses-reduction of ifosfamide was used in this trial (82).

In one retrospective study, RT (40Gy) in combination with the ESHAP (etoposide, steroid, high-dose Ara-C, and platinum) regimen were simultaneously delivered during a CCRT induction phase, followed by a consolidation phase with another 2-3 cycle of ESHAP chemotherapy alone. All the 13 patients responded effectively. At the end of the treatment, CR was achieved in 12/13 (92%) patients and with a median follow-up of 38 months, the 2-year OS rate reached 72%. However, 12 of the 13 patients experienced grade 3–4 hematological toxicity, and secondary malignancy was observed in 2 patients during follow up possibly due to RT (83).

A new intramaxillary arterial infusion chemotherapy of MPVIC-P (ifosfamide, carboplatin, methotrexate, peplomycin, and etoposide), was administered with concomitant local RT for early stage ENKL. Compared with systemic administration, intra-arterial chemotherapy showed higher drug concentrations and fewer complications. All of the 12 patients achieved CR, and their serum EBV-DNA copy numbers decreased to below the detectable levels (84).

Other protocols such as CCRT with MIDLE (methotrexate, etoposide, dexamethasone, and L-asparaginase) regimen (85), CCRT followed by VIDL (etoposide, ifosfamide, dexamethasone and L-asparaginase) (86) or LVDP (L-asparaginase, cisplatin, etoposide and dexamethasone) (87) or GDP (gemcitabine, dexamethasone and cisplatin) (88), have also demonstrated certain extent of curative effect for localized ENKL and are summarized in Table 2. Nevertheless, high disease progression or refractory rates and hematologic toxicity were commonly found in those studies.

**Table 2:** Selected clinical studies on the radiotherapy and chemotherapy of ENKL.

**Stage**	**Protocol**	**Study**	**No**.	**Treatment**	**Mid. follow-up, month**	**ORR (%)**	**CR (%)**	**OS%**	**PFS%**
I/II	Chemotherapy + RT + chemotherapy	Prospective (78)	26	RT with LVP	27	89	81	89% (2y)	81% (2y)
I/II	Chemotherapy + RT + chemotherapy	Prospective (79)	27	RT with GELOX	27	96	74	86% (2y)	86% (2y)
I/II	RT + chemotherapy	Retrospective (80)	44	RT + GDP	38	95	89	85% (3y)	77% (3y)
I/II	CCRT	Prospective (81)	27	RT + DeVIC	32	81	77	78% (2y)	NA
I/II	CCRT	Retrospective (65)	150	RT + DeVIC	67	89	82	72% (5y)	61% (5y)
I/II	CCRT + chemotherapy	Prospective (82)	30	CCRT + VIPD	NA	83	80	86% (3y)	85% (3y)
I/II	CCRT + chemotherapy	Retrospective (83)	13	CCRT + ESHAP	38	100	92	72%(2y)	90%(2y)
I/II	CCRT + chemotherapy	Prospective (86)	30	CCRT + VIDL	44	NA	87	73% (5y)	60% (5y)
I/II	CCRT + chemotherapy	Prospective (87)	66	CCRT + LVDP	24	86	83	70% (3y)	67% (3y)
I/II	CCRT + chemotherapy	Prospective (88)	28	CCRT + GDP	38	91	84	88% (3y)	84% (3y)
I/II	CCRT	Retrospective (84)	12	RT with MPVIC-P	–	100	100	–	–
Advanced and rel/ref	Chemotherapy	Prospective (89)	38	SMILE	24	79	45	55% (1y)	53% (1y)
Advanced and rel/ref	Chemotherapy	Prospective (90)	86	SMILE	31	81	66	50% (5y)	64% (4y)
Advanced and rel/ref	Chemotherapy	Retrospective (91)	13	MEDA	NA	77	62	69% (1y)	62% (1y)
Advanced and rel/ref	Chemotherapy	Retrospective (92)	24	P-GEMOX	28	80	51	39% (3y)	65% (3y)
Advanced and rel/ref	Chemotherapy	Retrospectively (93)	41	GDP	16	83	42	73% (1y)	55% (1y)

*Mid, median; ORR, overall response rate; CR, complete response; OS, overall survival; PFS, progression-free survival; RT, radiotherapy; CCRT, concurrent chemoradiotherapy; NA, not available; rel/ref, relapsed/refractory*.

### Advanced and Relapsed/Refractory NK/T-Cell Lymphoma

#### Chemotherapy

The current treatment consisting of non-anthracycline-based chemotherapy followed by or in combination with RT has improved the outcomes of localized ENKL patients. However, patients with advanced disease still face the risk of progression or relapse after initial treatment. And those relapsed/refractory patients always have unfavorable outcomes.

Several studies have identified significant benefit of L-asparaginase-containing chemotherapeutic regimens to clinical outcome in ENKL, and consider it as the current optimal treatment for salvage therapy (94–96). A novel L-asparaginase-containing chemotherapeutic regimen SMILE (dexamethasone, methotrexate, ifosfamide, L-asparaginase, and etoposide) has shown great efficacy and is recommended as the first-line therapy for both localized and advanced ENKL patients (97). In a phase II study enrolling 38 patients, in which 20 of them had newly diagnosed stage IV disease, 14 were in their first relapse, and 4 were in primary refractory state, were all prescribed the SMILE chemotherapy. After two cycles, the results showed an ORR of 79% with a CR rate of 45%. No differences were found in either the ORR or CR rate between patients with newly diagnosed stage IV disease and those with first-relapse disease. The major toxicity, Grade 4 neutropenia, was observed in 92% of the patients. After a median follow-up of 24 months, the 1-year OS and PFS rates were 55 and 53%, respectively (89). To identify the efficacy and safety of this regimen, 87 patients (43 newly diagnosed advanced patients and 44 relapsed/refractory patients) of an unselected cohort were treated with the SMILE regimen, demonstrated an ORR of 78% (81% of the advanced disease and 75% of the relapsed/refractory, respectively) in an interim analysis. After confirmation of CR at interim analysis, 19 patients received sequential RT. On completion of treatment, the ORR rate was increased from 66 to 81% (84% of the advanced disease and 77% of the relapsed/refractory, respectively). Significant toxicities included grade 3/4 neutropenia, grade 3/4 thrombocytopenia, and nephrotoxicity (90).

Another new chemotherapeutic regimen DDGP (cisplatin, dexamethasone, gemcitabine, and pegaspargase), was established for newly diagnosed advanced ENKL patients. Pegaspargase, an E.coli-derived L-asparaginase that has previously shown lower toxicity, was given to those patients as a substitution of L-asparaginase. In order to assess the efficacy and toxicity of the DDGP and SMILE regimen in newly diagnosed advanced ENKL, a randomized controlled, multicenter study was conducted in China. It indicated that DDGP improved efficacy and reduced toxicity compared with the SMILE group, the DDGP group demonstrated better ORR (95 vs. 67%), CR (71 vs. 29%), and PR (24 vs. 38%) rates. And the OS and PFS in the DDGP group were also significantly better than the SMILE group: 86 vs. 38% for the 1-year PFS and 74 vs. 45% for the 2-year OS, respectively. For the adverse effect, there were more instances of grade 3/4 leukopenia and grades 3/4 allergy in the SMILE group. However, grade 3/4 anemia was more common in the DDGP group than in the SMILE group (98).

Another L-asparaginase-containing regimen AspaMetDex (L-asparaginase, methotrexate, and dexamethasone) has also shown satisfactory result in relapsed/refractory patients. After 3 cycles of AspaMetDex chemotherapy, 11 of the 18 (61%) evaluable patients reached CR, and the median OS and PFS were both 12 months, respectively. Neutropenia, hepatitis, anemia, and allergy was common during the protocol (94). Moreover, the combination regimen of gemcitabine, oxaliplatin, and pegaspargase (P-gemox) was investigated in 35 patients with advanced, relapsed/refractory ENKL. On completion of the treatment, the ORR was 80%, with an observed CR in 51% of the patients. The major drug-related toxicity were hematologic toxicity and liver dysfunction. APTT elongation and hypoalbuminemia could also be frequently detected (92). Regimens like MEDA (methotrexate, etoposide, dexamethasone, and pegaspargase) (91) or GDP (gemcitabine, dexamethasone, and cisplatin) (93) have also shown efficacy for advanced, relapsed/refractory ENKL and details are shown in Table 2.

In conclusion, there are some chemotherapeutic options for advanced, relapsed/refractory patients, but no standard treatment has been established and the overall survival of advanced and relapsed/refractory patients is relatively poor. In consideration of all those adverse effects due to the current therapy, further research is required to validate the efficacy and tolerance in an expanded number of patients.

#### Hematopoietic Stem Cell Transplantation

Hematopoietic stem cell transplantation (HSCT) has shown excellent advantage in improving long-term survival of leukemia and lymphoma patients, whereas the experience regarding HSCT in ENKL is still limited (Table 3).

**Table 3:** Selected studies of autologous and allogeneic HSCT for ENKL patients.

**HSCT**	**Study** ** design**	**No**.	**First-line therapy**	**Disease stage**	**Mid. follow-up, month**	**OS%**	**PFS%**	**TRM%**
Autologous HSCT	Retrospective (99)	62	Noneanthracycline-based *n* = 51 (82%)	Localized (*n* = 31) Advanced (*n* = 31)	43	68 (3y) 52 (3y)	65 (3y) 40 (3y)	3.2
	Retrospective (36)	28	Asparaginase-containing *n* = 6 (21%)	Localized (*n* = 16) Advanced (*n* = 10) Unknown (*n* = 2)	33	52 (2y)	41 (2y)	11^*^ (1y) 22^*^ (2y)
	Prospective (100)	80	Asparaginase-containing *n* = 80	Autologous HSCT (*n* = 20) Controls (*n* = 60)	80	79 vs. 52%, *p* = 0.026 (5y)	NA	0
Allogeneic HSCT	Retrospective (101)	18	SMILE (78%)	Localized (*n* = 5) Advanced (*n* = 13)	21	57 (5y)	51 (5y)	22
	Retrospective (102)	82	SMILE (15%)	Localized (*n* = 35) Advanced (*n* = 22) Unknown (*n* = 25)	36	34 (3y)	28 (3y)	30^*^
	Retrospective (103)	12	NA	Advanced (*n* = 12)	16	55 (3y)	53 (3y^#^)	8.3

HSCT, hematopoietic stem cell transplantation; Mid, median; TRM, transplantation - related mortality;

* NRM, non-relapse Mortality; NA, not available;

# *EFS, event-free survival*.

According to the reported database of the Center for International Blood and Marrow Transplant Research (CIBMTR), the treatment data of 82 patients with ENKL who underwent allogeneic HSCT were first reviewed, and after a median follow-up of 36 months, their 3-year PFS and OS were 34 and 28%, respectively. Non-relapse mortality (NRM) and 3-year relapse rate were 30 and 42%, respectively. No relapses were observed after 2-year. Up to the last follow-up, lymphoma relapse or progression was the most common cause of death (102). Another study analyzed 18 patients with ENKL who underwent allogeneic HSCT. After a median follow-up of 21 months, the 5-year OS and 5-year event-free survival (EFS) was 57 and 51%, respectively. Pre-treatment with the SMILE regimen was observed as a positive prognostic indicator for superior OS and EFS. However, the occurrence of acute graft-vs.-host disease (GVHD) was correlated with a poor OS (101).

In a retrospective study enrolling 80 ENKL patients, 20 of them were given induction chemotherapy followed by autologous HSCT and the remaining 60 received induction chemotherapy, which were selected as the control group. The five-year OS between the two groups demonstrated a significant difference, 79 vs. 52%, respectively, suggesting that treatment of chemoradiotherapy with autologous HSCT improve the outcome of ENKL patients compared to that without autologous HSCT (100). In another study which included 62 patients who underwent autologous HSCT after primary therapy, the pre-transplant responses showed a CR of 61% and PR of 39%, and after the transplantation, the CR was increased to 78%. The 3-year PFS and OS were 52 and 60%, respectively. Patients with limited disease showed better 3-year PFS and OS than those with advanced disease (99).

The American Society for Blood and Marrow Transplantation recommend allogeneic HSCT as a consolidation for disseminated ENKL, and both allogeneic HSCT and autologous HSCT are recommended for relapsed-sensitive ENKL in localized as well as disseminated patients. However, any HSCTs are not recommended as a first-line therapeutic management for localized ENKL (104).

#### Immunotherapy

Outcomes of advanced and relapsed or refractory patients has been improved since the application of traditional L-asparaginase-containing chemotherapy, however, the CR rates still ranges between 50 and 60%, with short OS and PFS time. Thus, novel strategies are required to change the current treatment-outcome situation. The application of immune checkpoint blockades has proved to reduce the growth of a variety of tumor cells, and updates the treatment modality of current oncotherapy (Figure 2).

Programmed death receptor 1 (PD-1) is an inhibitory receptor expressed on the surface of activated T cells, and its ligand programmed death protein ligand 1 (PDL1) is found highly expressed in various tumor cells. Interaction of PD-1 with PDL1 inhibits T-cell activation and proliferation, and help tumor cells to escape from immune surveillance, thus making PD-1/PDL1 axis an immune checkpoint to suppress anti-tumor immunity (23). Therapies with antibodies targeting PD-1 and its ligands have shown satisfactory outcomes in various cancers. As for ENKL, pembrolizumab, an anti-PD1 antibody, was tested in 7 relapsed patients who failed to respond to previous L-asparaginase regimens. After a median of 7 cycles of the treatment, 5 of the patients achieved CR and 2 of the patients achieved PR. The ORR was 100%, showing a high sensitivity to anti-PD1 antibody (105). Another 7 patients with ENKL who failed upon treatment with previous chemotherapies were also treated with pembrolizumab, but only 4 patients responded (CR = 2, PR = 2, 57%), even though, the result was still considered as satisfactory (106). Nivolumab, another anti-PD1 antibody, also showed efficacy in clinical practice. Toxicity typically associated with anti-PD1 antibodies was not observed, but two patients developed grade 1 cytokine release syndrome and tumor lysis syndrome due to high sensitivity to the drug (107).

Adoptive immunotherapy with LMP-specific cytotoxic T cells (CTLs) were designed to target LMP on ENKL cells (108). To evaluate the efficacy and safety of this treatment, 10 ENKL patients previously achieving CR were treated with LMP-1 specific CTLs. No immediate or delayed toxicities were observed. After a median follow-up of 55.5 months, the 4-year OS and PFS were 100 and 90%, respectively (109). Promising therapeutic options efficaciously inhibiting the release of TNF-α and IFN-γ like dexamethasone and thalidomide will be an important step to take immunosuppressive agents further into the treatment of *ECSIT-V140A*-associated NK/T-LAHPS (24). Anti-CD38 antibody, daratumumab, which has been approved for use in multiple myeloma patients, has also shown efficacy in ENKL patient. In one case, after failure to response to CCRT, asparaginase–based consolidation chemotherapy and allogeneic HSCT, a relapsed, refractory woman with ENKL was given daratumumab. After completion of the treatment, she achieved a remission lasting for 21 weeks. However, larger trials are still required for a more comprehensive assessment of the efficacy and safety of this strategy (110).

#### Other New Drugs

Identifying mutations that affect their functions is key to the development of targeted, personalized therapies. Notably, persistent activation of JAK-STAT3 resulting from activated mutations may evolve in the pathogenesis of ENKL, and deserves to be investigated as a potential therapeutic target.

JAK inhibitor, which interrupt JAK-STAT pathway, have shown satisfactory therapeutic efficacy *in vitro* with STAT3 and STAT5 mutated cells (35). Downstream effector proteins of phosphorylated STAT3 and STAT5 could be shapely downregulated through hyperactivated JAK3-STAT-mediated signaling cascade. CP-690550, a novel JAK3 inhibitor, was reported to result in a dose-dependent downregulation of phosphorylated JAK, to reduce cell growth and to induce apoptosis in JAK3-mutant ENKL cell lines (34). It inhibited the tumor cell growth in the human ENKL as well as xenograft mouse model harboring JAK3 activating mutation (33). Small-molecule inhibitor PRN371 was a highly selective JAK3 inhibitor. It showed longer-lasting effect compared with tofacitinib, which functioned as a pan-JAK inhibitor (50). Nowadays, JAK inhibitor that approved by FDA is available for clinical trials in ENKL patients, while STAT3 and STAT5B inhibitors are clinically available but still not approved by FDA. However, the efficacy of JAK/STAT inhibitors has not yet been verified in clinical trials. In addition, the pan-JAK inhibiting activity of tofacitinib and CP-690550 has limited their clinical utilization in cancer therapy.

Bortezomib, a potent and reversible proteasome inhibitor which has shown efficacy in preclinical models of ENKL *in vitro*, is under evaluation in a phase II trial. Seven newly diagnosed ENKL patients received bortezomib-GIFOX (gemcitabine, ifosfamide, oxaliplatin) regimen and achieved an ORR of 43%, although the median PFS was only ~4 months (51).

At present, drugs targeting of EZH2 show great potential for better treatment patterns of cancers. Data shown above indicates that targeting EZH2 may have therapeutic prospect in ENKL. GSK126, a highly selective inhibitor of EZH2, markedly inhibits the growth of EZH2 mutant DLBCL cell lines and DLBCL xenografts (52). Currently, more studies are needed to verified GSK126's efficacy in ENKL. Another new drug, romidepsin, a histone deacetylase (HDAC) inhibitor, failed to show favorable result in ENKL because of EBV's reactivation, thought it has been approved for relapsed or refractory peripheral T-cell lymphoma (111, 112).

## Conclusion and Prospect

In the last decades, the recognition and treatment outcomes for NK/T-cell lymphomas have shown notable achievements. Since the identification of genetic alterations has shed light on the molecular pathogenesis of this disease, potential targeted specific strategies are to be established for clinical application.

Non-anthracycline and L-asparaginase containing chemotherapy regimens have devoted predominant efforts in the current ENKL treatment. Immune checkpoint inhibitors and small-molecule inhibitors have also shown bright prospect as future therapeutics. Patients who demonstrate a CR to treatment have been observed to have better PFS than those who did not, reversely, those who failed to reach CR may face multiple drug resistance and a shorter survival time. How to choose the optimal regimen and how to improve long-term survival of ENKL patients are still challenges that are to be fully addressed, and thus, continued study are urged to identify better pathogenic mechanism, prognostic factors, and novel strategies.

## Author Contributions

QC, JC, YF, and KY wrote and approved the final version of this manuscript.

### Conflict of Interest Statement

The authors declare that the research was conducted in the absence of any commercial or financial relationships that could be construed as a potential conflict of interest.
